# ABC Transporters in *Dictyostelium discoideum* Development

**DOI:** 10.1371/journal.pone.0070040

**Published:** 2013-08-14

**Authors:** Edward Roshan Miranda, Olga Zhuchenko, Marko Toplak, Balaji Santhanam, Blaz Zupan, Adam Kuspa, Gad Shaulsky

**Affiliations:** 1 Department of Molecular and Human Genetics, Baylor College of Medicine, Houston, Texas, United States of America; 2 Graduate Program in Developmental Biology, Baylor College of Medicine, Houston, Texas, United States of America; 3 Department of Biochemistry and Molecular Biology, Baylor College of Medicine, Houston, Texas, United States of America; 4 Faculty of Computer and Information Science, University of Ljubljana, Ljubljana, Slovenia; 5 Graduate Program in Structural and Computational Biology and Molecular Biophysics, Baylor College of Medicine, Houston, Texas, United States of America; University of Cambridge, United Kingdom

## Abstract

ATP-binding cassette (ABC) transporters can translocate a broad spectrum of molecules across the cell membrane including physiological cargo and toxins. ABC transporters are known for the role they play in resistance towards anticancer agents in chemotherapy of cancer patients. There are 68 ABC transporters annotated in the genome of the social amoeba *Dictyostelium discoideum*. We have characterized more than half of these ABC transporters through a systematic study of mutations in their genes. We have analyzed morphological and transcriptional phenotypes for these mutants during growth and development and found that most of the mutants exhibited rather subtle phenotypes. A few of the genes may share physiological functions, as reflected in their transcriptional phenotypes. Since most of the *abc*-transporter mutants showed subtle morphological phenotypes, we utilized these transcriptional phenotypes to identify genes that are important for development by looking for transcripts whose abundance was unperturbed in most of the mutants. We found a set of 668 genes that includes many validated *D. discoideum* developmental genes. We have also found that *abcG6* and *abcG18* may have potential roles in intercellular signaling during terminal differentiation of spores and stalks.

## Introduction

ABC-transporters belong to one of the largest superfamilies of transporters in cells of all kingdoms [1]. *abc* genes encode conserved multispan trans-membrane proteins that export cargo molecules while utilizing ATP hydrolysis. They participate in multiple pathways by exporting a wide variety of molecules, including lipids, peptides, amino acids, carbohydrates, ions, and xenobiotics [2]. ABC proteins can be found on multiple membranes, including the plasma membranes and membranes of intracellular compartments such as the Golgi, endosome, multi-vesicular bodies, endoplasmic reticulum, peroxisome, and mitochondria.

The *D. discoideum* genome contains 68 *abc* genes, which have been classified into 8 groups, *abcA* through *abcH*, based on their sequence and structural features [3], [4]. A fully functional ABC-transporter contains two nucleotide-binding domains and two 6-pass trans-membrane domains. The ABCA, ABCB, ABCD, and ABCG classes of transporters include a few half-transporters, which contain one nucleotide-binding domain and one 6-pass trans-membrane domain. Such half-transporters may form hetero- or homo-dimers that function as full transporters. ABC-transporters of the classes ABCE, ABCF, and ABCH, do not contain any predicted trans-membrane domains and hence may not function as transporters or may need to associate with other proteins to function as transporters [4]. There is currently no general functional classification of these genes because the majority of transporters has not been characterized genetically or functionally in any given organism.

Whole genome expression profiles, obtained by RNA-seq or microarrays, can be used as molecular phenotypes for genome-wide functional analysis [5]–[10]. Transcriptional phenotypes provide quantitative parameters that can be used to compare between mutants, identify networks of gene interactions, perform functional analysis of genes, and even analyze epistatic relationships [5]–[7]. Transcriptional response phenotypes are considered as higher resolution phenotypes than morphological phenotypes, especially where morphological phenotypes are less diverse and hence less informative [7]. RNA-seq has recently supplanted microarrays as the method of choice to obtain global transcriptional responses to environmental changes or mutations [6], [11]. The addition of multiplexing resulted in considerable reduction in cost [12], [13]. In this study we used transcriptional phenotyping with RNA-seq to infer functional similarities between different *abc* transporter mutants in *D. discoideum* and to identify genes that are important for development.

*D. discoideum* are social amoebae that grow as solitary cells when food is abundant and embark on multicellular development when food is scarce. Upon starvation, around 10^5^ cells aggregate through a process of chemotaxis to form a mobile structure called a slug, which ultimately becomes a fruiting body. The fruiting body is comprised of 80% spores that can germinate and propagate to the next generation, and 20% stalk cells, which die while supporting the spore cell mass [14]. *Dictyostelium* development involves multiple signaling molecules and many genes that process them [15], including the *abc* transporter genes *tagB*, *tagC* and *tagA* [16], [17].

In this study, we have obtained mutants for 35 of the *abc*-transporter genes in *D. discoideum* and characterized them based on their morphological and transcriptional phenotypes. We observed that most of the mutants exhibited subtle morphological phenotypes, but the higher resolution of their transcriptional phenotypes allowed us to group them into functional groups. We have also utilized the transcriptional phenotypes to identify genes that are important for *D. discoideum* development. We further show that *abcG6* and *abcG18* influence spore differentiation during the final stages of development.

## Materials and Methods

### Strain construction, growth and development

The *abc* transporter genes were mutated by homologous recombination in the parental strain AX4. We used two methods to construct the DNA vectors for mutating the genes: a) transposon mutagenesis [18] and b) direct cloning [16]. The vectors and the *Dictyostelium* strains are described in Table S1. We verified the insertion sites in each mutant by performing PCR on genomic DNA and also validated each strain by RNA-seq, testing that the insertion modified the expression of the respective *abc* gene in each case.

We grew all the strains in liquid suspension in HL5 medium supplemented with streptomycin (50 mg/mL) and penicillin (50 U/mL), shaking at 220 rpm at 22°C [19]. For scoring morphological phenotypes, we harvested the cells at the logarithmic growth phase by centrifugation and washed them once with KK2 buffer (16.3 mM KH_2_PO_4_, 3.7 mM K_2_HPO_4_, pH 6.2). We deposited 5×10^7^ cells onto a 5 cm nitrocellulose membrane placed on a pad saturated with 2 mL PDF buffer (9.2 mM K_2_HPO_4_, 13.2 mM KH_2_PO_4_, 20 mM KCl, 1.2 mM MgSO_4_, pH 6.5). We incubated the cells at 22°C in a dark humid chamber [19]. We recorded the developmental phenotypes at each time point as specified in the text. Terminal phenotypes were recorded 48 hours after the initiation of development.

### Sporulation efficiency

We grew and developed 2.5×10^7^ cells on a 5 cm nitrocellulose membrane as described above. We harvested the entire population after 48 hours into a 50 mL falcon test tube containing 10 mL of KK2 buffer, 10 mM EDTA, and 0.1% NP40. We passed the cell suspension 10 times through an 18G syringe needle and counted the spores under a microscope. To test whether sporulation defects were cell autonomous, we mixed mutant and GFP-labeled wild-type cells at equal proportions and developed them together as above. After 48 hours of development, we counted the visible non-fluorescent spores (mutant) and green-fluorescent spores (wild type) as described [20]. Briefly, green fluorescence was normalized to the number of GFP-labeled wild-type spores that were fluorescent when developed in a pure population (usually greater than 90%) and in a 1∶1 mix with unlabeled wild-type cells. Each experiment was repeated twice unless otherwise indicated. Average values or actual values are presented in Table 1.

**Table 1:** Morphological phenotypes, sporulation efficiencies and sorting preferences of all the *abc* mutant strains during development.

Mutant	Doubling time (h)	Morphological phenotype					Terminal phenotype	Sporulation efficiency (%)	Sorting preference in chimera with WT	Devel. category
		0 h	6 h	12 h	18 h	24 h				
A3	9.0	VEG^a^	RIP	LAG, MND	MND, FNG, SLG	MND, FNG, FBS	MND, FNG, GNR	73±2.0	none	Normal
A4	8.9	VEG	RIP	TAG, MTAG	TAG, MFNG, SLG	MND, FNG, ECUL, FBS	GNR, FBS	91±4.0	none	Normal
A5	10.3	VEG	RIP	MND	FNG, SLG, MND	FBS, ASN	ASN	32±6.7	prestalk (pstO)	Normal
A6	11.5	VEG	RIP	TAG, MTAG	MFNG, SLG, MXH	SFBS	SFBS	ND	ND	Normal
A7	10.2	VEG	RIP	TAG	FNG, MXH	FBS, ASN	ASN	50, 70	prespore	Normal
A9	8.5	VEG	RIP	TAG, MTAG	FNG, MFNG, SLG	FBS, ASN	FBS, GNR	45, 60	none	Normal
A10	11.4	VEG	RIP	MND, TAG	MFNG, SLG	FNG, SLG, ECUL, ASN	FBS, ASN	53, 55	prestalk (pstA)	Normal
A11	7.7	VEG	RIP	LAG, MND	ECUL, FNG, SLG, MXH	FNG, SLG, FBS, ASN	FBS	58, 78	prestalk (pstO)	Normal
B1	7.2	VEG	RIP	TAG	TAG, SLG, ECUL, MXH	ECUL	LFB	93±8.6	prestalk (pstO)	Normal
B4	8.4	VEG	RIP	TAG	ECUL, MXH, FNG	FBS, MFBS	EFB, MFNG	38, 48	prestalk (pstO)	Normal
B5	15.3	VEG	RIP	TAG, FNG	SLG, FNG	ECUL	LFB	82, 117	prestalk (pstO)	Normal
C2	11.4	VEG	LAG	MTAG	MFNG	MFBS, MFNG	MFBS, ASN, LFB	79±3.1	prestalk (pstO)	Normal
C3	11.8	VEG	RIP	MTAG, TAG	MFNG, FNG, MXH	SLG	LFB	53, 80	prespore	Normal
C6	10.0	VEG	RIP	LAG, TAG	SLG, FNG	FBS, ECUL, MND	ASN, LFB	ND	ND	Normal
C8	18.9	VEG	RIP	LAG	MFNG, FNG	MFBS	MFBS	80±1.6	prespore	Normal
C12	11.0	VEG	RIP	TAG	ECUL, FNG	EFB	FBS	66, 81	prestalk (pstA)	Normal
C13	9.9	VEG	RIP	TAG, MTAG	TAG, FNG, SLG, ECUL	ECUL	LFB, ASN	50, 51	prestalk (pstO)	Normal
C14	8.4	VEG	RIP	TAG, MTAG	FNG, MXH, SLG	SFBS, ASN	SFB, ASN	38, 50	prestalk	Normal
D2	11.2	VEG	RIP	MND	FNG	SFBS	SFBS	90, 97	prestalk (pstO)	Normal
F1	7.0	VEG	RIP	TAG	TAG, SLG, ECUL	ECUL, ASN	ASN, LFB	80±6.0	prestalk	Normal
F2	8.3	VEG	RIP	MND	TAG, FNG	ECUL	FBS	37, 47	none	Delayed
F4	7.3	VEG	RIP	TAG, MTAG	FNG, SLG, MFNG, ECUL	ECUL, FBS, ASN	FBS, ASN, GNR	65, 85	prestalk (pstO)	Normal
H2	9.7	VEG	RIP	MTAG	MFNG	FNG, ECUL, LCUL	FBS	0, 0	ND	Normal
G2	10.8	VEG	RIP	LAG, STR	TAG, FNG	ECUL, SFBS	SFBS	50, 88	prestalk (pstA)	Delayed
G5	9.9	VEG	RIP	TAG, FNG	SLG	ECUL	LFB	73±4.3	prestalk (pstO)	Normal
G6	8.4	VEG	RIP	TAG, MTAG	FNG, SLG, MFNG	FBS	FBS	58±12.6	none	Normal
G7	10.4	VEG	RIP	LAG, MND	TAG, FNG	ECUL, SFBS	SFBS	13, 25	prestalk (pstO)	Delayed
G10	9.1	VEG	RIP	STR, RIP	TAG, FNG	ECUL, LCUL, SFBS	SFB	10, 50	prestalk	Delayed
G15	8.4	VEG	RIP	TAG	FNG, SLG, TAG	FBS, MXH	ASN	32, 40	prespore	Normal
G16	11.5	VEG	RIP	TAG	FNG, SLG, MXH	SLG, MXH	LFB	66, 110	none	Normal
G17	9.7	VEG	RIP	TAG	SLG, MXH	SFBS	SFB	19±2.2	prestalk	Normal
G18	8.3	VEG	RIP	MND, LAG	TAG, FNG	MFBS	MFNG	80±8.1	prestalk	Normal
G19	8.0	VEG	RIP	RIP	RIP	RIP	SFBS	ND	prestalk	No development
G22	9.3	VEG	RIP	STR, RIP	TAG, FNG	TAG, SLG, ECUL, FBS, ASN,	FBS, GLR, ASN	19, 28	none	Delayed
G24	8.5	VEG	RIP	TAG	TAG	FNGS, ECUL	LFB	6, 18	prestalk	Normal
WT	8.0	VEG	RIP	TAG	SLG	FBS	FBS	113±5.0		

a Abbreviations: ASN = asynchronous; CUD = culmination defective; ECUL =  Early culminants; EFB = early fruiting bodies; FBS = fruiting bodies; FNG = fingers; LAG = loose aggregate; LCUL = late culminants; LFB = late fruiting bodies; MFBS = Multiple fruiting bodies from a single base; MFNG = Multiple fingers; MND = mounds; MTAG = Multi tipped aggregate; MXH = Mexican hats; RIP = Ripples; SFBS = small fruiting body; SLG = Slugs; STR = streams; TAG = tipped aggregate; VEG = Vegetative; ND: not determined. The morphologies expected during wild type development are provided in Table 2.

### Transcriptional responses of *abc* genes

To determine the transcriptional response of *abc* genes to various treatments at the vegetative stage, we grew wild-type cells in 5 mL of shaking culture in the presence of 5 mM cAMP, 100 nM DIF, or 10 µM Cisplatin in HL5 medium for 16 hours. We extracted RNA and prepared cDNA using the Cell-to-cDNA II kit (Ambion, TX, USA) according to manufacturer's recommended protocol. We determined the abundance of each *abc* transporter transcript by quantitative RT-PCR using oligonucleotides specific to each *abc* transporter gene (Table S2). Treated samples were compared to untreated samples; we considered transcripts that exhibited a twofold change between the treated and untreated samples as differentially responsive. To determine the transcriptional response of *abc* genes to various treatments during development, we developed 1×10^7^ cells on 5 cm petri dishes containing 1.5% agar in KK2 buffer supplemented with 5 mM cAMP or 100 nM DIF-1. We prepared cDNA from cells at the slug stage (around 20 hours of development) and performed quantitative RT-PCR using *abc*-gene-specific oligonucleotides.

### Sorting assays

To determine the position of the mutant cells in chimeric slugs, we mixed unlabeled mutant cells and GFP-labeled wild-type cells [21] at equal proportions (1∶1) and developed them together as above on 5 cm petri dishes containing 1.5% agar in KK2 buffer. We recorded the positions of the mutant cells in the slugs by fluorescence microscopy at around 20 hours of development.

### RNA purification and cDNA synthesis

RNA purification and cDNA synthesis were performed as described [22]. Briefly, we developed 5×10^7^ cells on a 5 cm nitrocellulose membrane as described above for the number of hours specified in the text. At each time point, we harvested the cells directly into 1 mL of Trizol® (life technologies, CA, USA) and extracted total RNA according to the manufacturer's recommended protocol. We performed two rounds of poly-A selection and fragmented 100 ng of the resulting mRNA into approximately 200 bases fragments. We prepared cDNA and the second strand as described before [6], [22].

### Illumina multiplexed library preparation for sequencing

We prepared Illumina multiplexed libraries as described [22]. We prepared 24 individual libraries separately (one from each sample) and added a unique barcode to each library at the final step of PCR amplification. We pooled equal amounts of DNA from each library and sequenced one pool per lane of a flow cell on an Illumina Genome Analyzer II using the manufacturer's recommended pipeline (versions 1.2 and 1.3, read length = 50 bases). The resulting FASTQ files were mapped using the short-read alignment software bowtie (version 0.12.7) allowing 2 mismatches. Mapping was performed as described [22]. The RNA-seq data are available at the NCBI GEO database, under accession number GSE45555 http://www.ncbi.nlm.nih.gov/geo/query/acc.cgi?token=xbuzhssekieyyxm&acc=GSE45555. Access to individual datasets and a complete list of the RNA-seq data are available in Table S3. We define the raw abundance level of a transcript as the sum of all the reads that uniquely map to that transcript. In order to compare transcript abundance between different samples, we normalized the raw abundance values to account for differences in the total number of mapable reads obtained with each RNA-seq run and differences in mapable gene length as described [22]. Briefly, we computed Exp_RPKM_ for every gene in the genome as the number of raw reads scaled per kilobase of the uniquely mapable length of exons per million uniquely mapped reads for the polyadenylated gene models in the experiment.
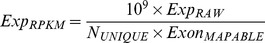
Where:

Exp_RAW_ = the number of raw reads for the gene

Exon_MAPPABLE_ = uniquely mapable length of the exons of the gene (nucleotides)

N_UNIQUE_ = total number of all uniquely mapped reads to polyadenylated gene models in the experiment

We used the Exp_RPKM_ values for all of the analyses, unless otherwise specified.

### Multidimensional scaling (MDS)

We computed the mean values of the transcript abundance for each gene at each time point and computed the distances between the transcriptional profiles of the different strains as 1 – Spearman's rank correlation coefficient. Multidimensional scaling (MDS), as implemented in Orange [23], was used to present the distances between all the *abc* mutants and the wild type.

### Differentially-expressed genes

We carried out differential expression analysis using the baySeq Bioconductor R package (http://www.bioconductor.org/packages/2.11/bioc/html/baySeq.html) [24]. First, we categorized the mutants as described in the text. We then used baySeq to compare genes in each category to the wild type in order to obtain a set of genes that were not differentially expressed (NDE) and a set of genes that were differentially expressed (DE). baySeq was parameterized using a bootstrap value of 3 and a convergence value of 1e-5 and two models were defined, NDE and DE. Genes with an FDR <0.005 and a Likelihood >0.90 were considered NDE.

## Results and Discussion

### *abc*-transporter mutants show robust developmental phenotypes compared to the wild type

**Figure 1:**
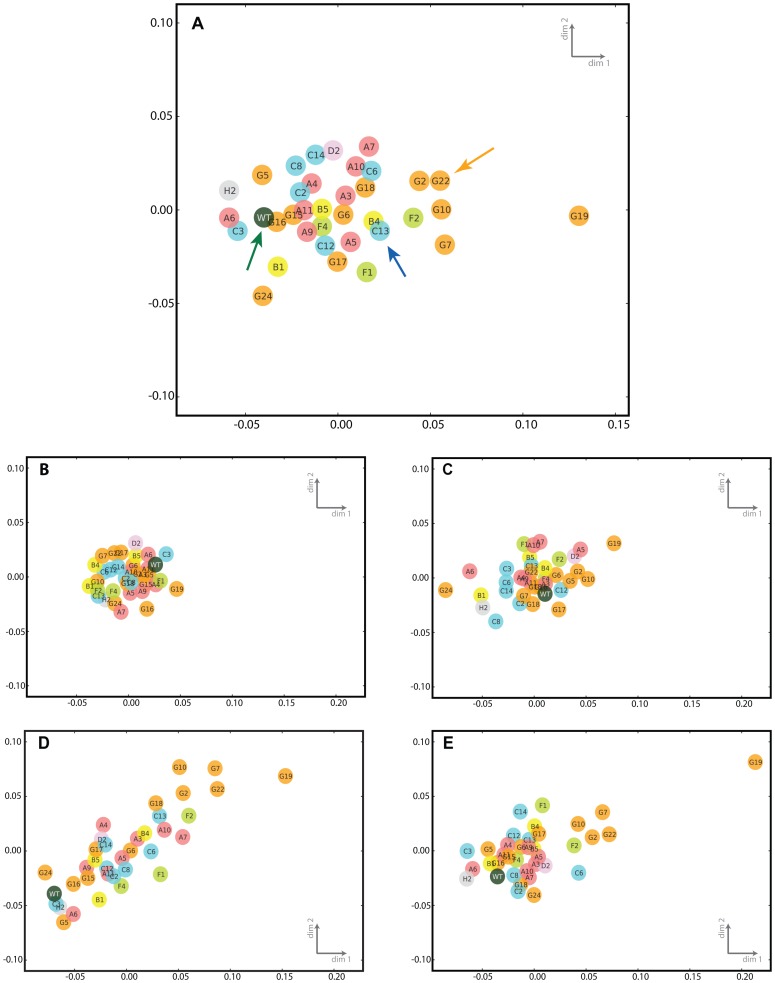
Transcriptional phenotypes of *abc*-transporter mutants. We determined the transcriptional phenotypes of the wild-type AX4 strain (WT) and 35 *abc*-transporter mutant strains at 0-, 6-, 12- and 18-hours of development. The relations between the transcriptional phenotypes are illustrated using multidimensional scaling, where the strains appear close together if their phenotype distance is small, or appear apart from each other if the phenotype distance is large. We constructed multidimensional scaling plots by considering transcriptional profiles for all the time points together where transcriptional profiles of each mutant at 0-, 6-, 12- and 18-hours were concatenated prior to distance computation (A) and for individual time points, 0-hours (B), 6-hours (C), 12-hours (D), and 18-hours (E). The axes are dimensionless. Each circle represents a strain, with an abbreviated strain name inside (e.g. G19 stands for *abcG19* ^−^). The colors indicate classes of ABC transporters. Arrows point to specific strains that are similar: tan – mutants showing delayed development, blue – *abcB4^−^ and abcC13^−^*, green – wild type.

We obtained transcriptional profiles for 35 *abc*-transporter mutant strains and for the laboratory wild-type AX4 strain in two biological replicates at four different time points during development. We developed the cells and collected RNA at 0-, 6-, 12- and 18-hours of development. We subjected the samples to RNA sequencing (RNA-seq), computed the distances between the transcriptional profiles and used multidimensional scaling (MDS) to visualize them in two dimensions (Figure 1). We surmise that close similarities in mutant transcriptomes suggest that the mutated genes have common physiological functions [7]. We also monitored the morphological phenotypes and sporulation efficiencies of all the strains during development and measured the transcript abundance of all the *abc*-transporter genes in wild-type cells under various conditions. Loss of one of the *abc* genes (*abcG19*) resulted in a severe delay in morphogenesis. Mutations in 4 other *abc* genes (*abcF2, abcG2, abcG7, and abcG10*) resulted in a delay in forming tipped aggregates. Mutations in 12 *abc* genes reduced the efficiency of sporulation to less than half that of the wild type. These results are summarized in Table S4 and Table S5.

We observed that most of the mutants showed similar transcription profiles to the wild type, except for the *abcG19^−^* mutant profile that was separated from the rest (Figure 1A). The *abcG19^−^* cells do not aggregate until 24 hours of development and form tiny fruiting bodies after 48 hours, suggesting that the transcriptional profile provides a valid reflection of the developmental process. In fact, the *abcG19^−^* profile became exceedingly different from the wild type over the course of development (Figure 1B–E), consistent with its attenuated development.

All the mutants exhibited transcriptional profiles that were very similar to the wild type at 0-hours (Figure 1B), which is consistent with the observation that their growth rates do not vary significantly (Table 1). As development progressed to 6-hours (Figure 1C), the similarity was reduced and an even greater dispersion was observed at 12-hours (Figure 1D). The transcriptional patterns became more similar to the wild type again at 18-hours of development (Figure 1E). However, we did not observe any clear trends in the dispersion of the morphological phenotypes as a function of time. This observation is consistent with the notion that the resolution obtained with transcriptional profiling is higher than the resolution of morphological phenotyping. The variation seen in the 12-hour time point might reflect greater asynchrony between the cells in the population at that time, but we do not have other data that could further validate that hypothesis. The *abc* genes have been classified according to their predicted amino acid sequences and predicted structures [4]. We colored the symbols in Figure 1 according to that classification, but we did not observe any class dependent similarities among the *abc*-transporter mutants.

These results support the idea that the transcriptional profiles provide a faithful representation of the mutant phenotypes and suggest that the differences observed between the mutants are informative. Moreover, it is curious that mutations in more than half of the *abc*-transporter genes in *D. discoideum* have such small effects on growth and development.

A group of mutants that were similar to each other and were distant from the wild type includes *abcF2^−^*, *abcG2^−^*, *abcG7^−^*, *abcG10^−^* and *abcG22^−^* (tan arrow, Figure 1A). This divergence from AX4 is not evident at the beginning of development (Figure 1B). It begins to appear at 6-hours (Figure 1C) and becomes more evident at 12- and 18-hours of development (Figure 1D and E, respectively). Examination of the morphological phenotypes revealed that these mutants were delayed during later stages of development (Table 1 and Table 2). These results further suggest that the changes in developmental phenotypes are reflected in the transcriptional phenotypes. It is interesting that although 5 of 6 mutants that display delayed developmental phenotypes belong to the *abcG* class, they do not show other phenotypic similarities, suggesting that they do not have common functions.

**Table 2:** Morphological phenotypes during wild type development.

Time (h)	0	6	8	10	14	16	18	20	22	24
WT morphology	VEG	RIP	STR	LAG	TAG	FNG	SLG	MXH	EFB	FBS
Alternative morphology								CUL		

Abbreviations: VEG = Vegetative; RIP = Ripples; STR = streams; LAG = loose aggregate; TAG = tipped aggregate; FNG = fingers; SLG = Slugs; MXH = Mexican hats; CUL = culminant; EFB = early fruiting bodies; FBS = fruiting bodies.

**Figure 2:**
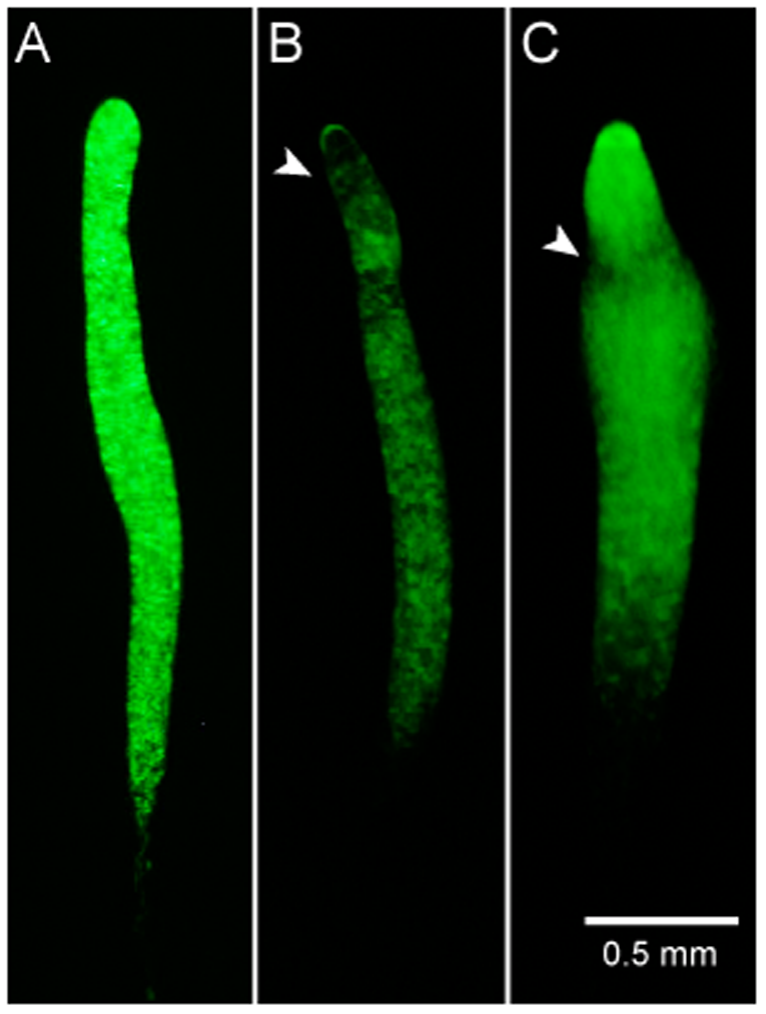
Sorting preference of the *abcB4^−^* and *abcC13^−^* mutants in chimeric slugs. We co-developed unlabeled mutant cells with GFP-labeled wild-type cells. Pictures were taken at 16-hours of development. In chimeric organisms, the wild-type cells (A) were evenly distributed throughout the slug; the *abcB4^−^* (B) and *abcC13^−^* (C) mutant cells preferentially sorted to the prestalk area and formed most of the pstO cells (arrowheads). Panels A and B are representative images whereas panel C represents fewer than half of the chimeric slugs observed.

Other similarities in the transcription profiles of the mutants were also correlated to morphological or other phenotypes (Table 1 and Table S4). Mutants *abcB4^−^ and abcC13^−^* (blue arrow, Figure 1A) displayed asynchronous development with similar levels of sporulation efficiency. In chimeric organisms, both mutant strains preferentially sorted to the prestalk area and formed most of the pstO cells when co-developed in a mix with GFP-labeled wild-type cells (Figure 2), although the sorting phenotype of the *abcC13^−^* strain was less penetrant and was observed in fewer than half of the slugs whereas the sorting phenotype of the *abcB4^−^* strain was observed in most of the chimeric slugs. Examination of the morphological phenotypes alone (Table 1) would have not revealed the similarities between *abcB4^−^ and abcC13^−^*.

The mRNA levels of most *abc*-transporter genes are developmentally regulated (Figure S1). We tested the correlation between the time of *abc*-transcript abundance in the wild type and the time of maximal deviation between the respective mutant phenotype and the wild-type phenotype. We found that for 18 mutants the time of peak transcript abundance preceded the time of maximal deviation and it overlapped with the time of maximal deviation for 8 mutants (Table 3). This finding suggests a causative relationship between the time of maximal transcript abundance and the time of maximal deviation from the wild-type phenotype, which is likely to reflect the time at which the respective genes function.

**Table 3:** Correlations between *abc*-transcript abundance and *abc*-gene function.

Peak transcript abundance relates to maximal deviation in phenotype	Precedes	Equals	Succeeds	No correlation
*abc* mutants	A3, A7, A9, B1, C2, C6, C8, C13, C14, D2, F1, F2, F4, G10, G15, G17, G19, G22	A6, A10, A11, B5, C12, G2, G7, G24	A4, A5, B4, G6, G18	C3, G5, G16, H2
Total number of mutants	18	8	5	4

A majority of the *abc*-transporter mutant strains showed decreased fitness levels, as evident from their low sporulation efficiencies and/or other aberrations in their development (Table 1), although all the *abc*-transporter mutants we analyzed were ultimately able to form fruiting bodies. These results suggest that the ABC transporters studied here are necessary for proper development even though the mutant strains do not exhibit extreme developmental defects under the conditions tested.

In higher organisms, mutations in *abc*-transporter genes that encode multi drug resistance (MDR) transporters do not result in overt phenotypes unless the mutant cells are challenged with toxins [25]–[27]. Considering these reports, we speculate that some of the *Dictyostelium abc*-transporter genes, which show peak transcript abundance at 0-hours, such as *abcA7*, *abcA9*, *abcB1*, *abcC2*, *abcC14*, and *abcG15*, may be involved in toxin resistance during cell growth. Few ABC transporters have also been implicated in developmental processes of other organisms [26], [28], [29]. Most of the *abc*-transporter mutants in our study show no strong developmental defects even though the respective genes are developmentally regulated. It is therefore unlikely that all of the remaining 33 transporters, which we have not characterized in this study, would have developmental defects if mutated. We speculate that the absence of overt developmental phenotypes is due to functional overlaps between ABC transporters. This possibility could be tested experimentally by characterizing the developmental phenotypes of strains in which two or more *abc*-transporter genes are mutated. Our results may be instructive in selecting small groups of *abc*-transporter genes for such analyses.

### Identification of developmental genes

**Figure 3:**
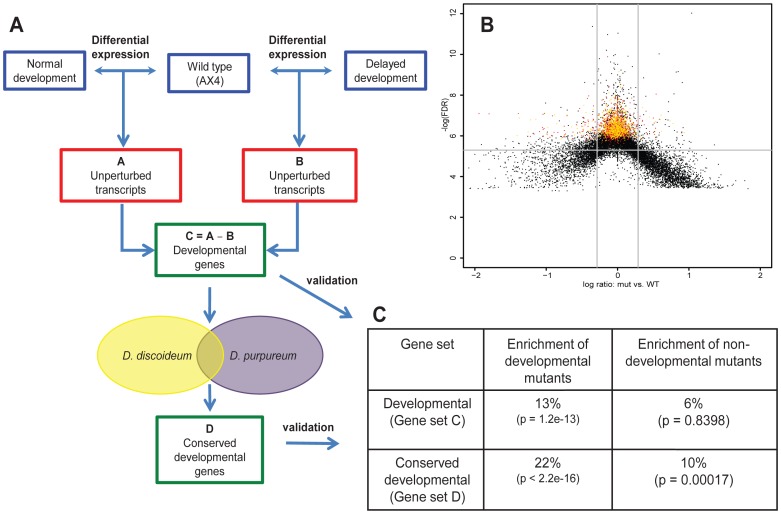
Identification of important developmental genes. The method used to identify developmental genes is depicted as a flow chart (A). Blue boxes represent the wild type (AX4) and mutant strains with normal or delayed development as indicated. Red boxes represent transcripts whose abundance was not significantly altered by the mutations. Green boxes represent selected developmental genes. Ellipses represent the genomes of *D. discoideum* (yellow) and *D. purpureum* (purple) and the overlapping orthologs (not to scale). The volcano plot (B) shows the false discovery rate (FDR) of each transcript (y-axis, −log[FDR]) as a function of the difference in mRNA abundance between the median of all the normally developing mutants and the wild type (x-axis, log(mutant/WT) at 12-hours, when the gene expression was most variable across mutants. Each dot represents a gene, yellow dots represent genes that were identified as important for development and conserved between *D. discoideum* and *D. purpureum* (Gene set D), red dots represent genes that show little or no perturbation in expression during development (Gene set C – Gene set D), and black dots represent the rest of the genes. We validated the approach by examining the enrichment of developmental and non-developmental genes in gene sets C and D (C).

Because most of the *abc*-mutants showed only subtle developmental defects, we hypothesized that the expression of genes crucial for development was not perturbed much in these mutants. To test this hypothesis and to identify a set of developmental genes, we divided the *abc*-transporter mutants into three categories, based on their phenotypes at the 12- and 18-hour time points. The first category included mutants that showed normal development, the second category included mutants that showed delayed development and the third category consisted of mutants that did not develop (only *abcG19^−^*) (Table 1). We did not consider the transcriptional responses at 0-hours because the mutants were nearly indistinguishable during growth (Table 1). We compared the transcriptomes of the mutants in each category to the wild-type transcriptome at 6-, 12- and 18-hours using baySeq [24]. In each comparison we identified sets of transcripts that showed little or no difference in abundance (FDR <0.005; Likelihood >0.90). We generated two sets of unperturbed transcripts, A – transcripts unperturbed in the normal development group and B – transcripts unperturbed in the delayed development or no development groups. We then excluded the genes in group B from the genes in group A to identify genes that were unperturbed exclusively in strains that developed normally. We named that group gene set C ({Gene set C} = {Gene set A} \ {Gene set B}) (Figure 3A). Gene set C includes 1,935 genes (Table S5).

Previously we found that the two evolutionarily divergent dictyostelid species, *D. discoideum* and *Dictyostelium purpureum* have conserved regulation of developmental gene expression. The set of 3,733 orthologous genes that exhibit conserved mRNA abundance patterns between these two species also shows enrichment in genes which, when mutated, confer developmental defects [6]. We found that 668 of the 1,935 genes in Gene set C belong to that group. We named that subset Gene set D (Table S6). We hypothesize that Gene sets C and D include genes that are important for *D. discoideum* development with higher enrichment in Gene set D. Comparing global mRNA abundance in mutants that developed normally and mRNA abundance in the wild type revealed the widest variability at 12-hours (Figure 3B), consistent with the observations made using MDS (Figure 1B–1D). However, the 668 genes in Gene set D were the least variable at that time (yellow dots, Figure 3B), suggesting that their regulation is largely unperturbed in the mutants.

To test whether Gene sets C and D are enriched in developmentally important genes, we looked for enrichment of genes with known developmental roles among these sets (Figure 3C). A list of genes that have essential developmental roles was obtained from dictyBase [30]. These genes constitute about 8% of the 12,285 genes in the *D. discoideum* genome. We found that Gene set C contained 13% and Gene set D contained 22% developmental genes. Both values were highly statistically significantly enriched compared to the overall level of 8% (Figure 3C). Gene set D includes genes that have been studied previously and their developmental mutants are well characterized, including *pkaC*, *tagC*, and *culB* [17], [31], [32]. It also includes 14 transcription factors, including *dimB*, *gbfA*, *crtf*, *warA*, and *sir2E*, which have important roles in development [33]–[37].

To test if the enrichment was specific to developmental genes, we also looked for enrichment of genes in which mutations do not confer obvious developmental defects. There are 620 such genes in dictyBase, which constitute 5% of the *D. discoideum* genome. We observed no significant enrichment in Gene set C and a modest enrichment in Gene set D (Figure 3C), suggesting that our method of differential expression analysis is predictive of developmentally important genes.

In this analysis we used mutants with subtle phenotypes, which are due to mutations in paralogous *abc* genes that may be involved in various pathways. This approach allowed us to find transcripts that show low plasticity despite the genetic perturbations we introduced. Efforts have previously been made to identify genes that are critical in processes such as cancer or other conditions by using meta-data to identify common sets of genes that vary in multiple conditions [38], [39]. Here, we used a counter approach and looked for genes that show the least variation upon subtle perturbation. The assumption in our analysis is that an alteration in mRNA abundance of developmentally important genes would lead to developmental abnormalities. This idea may explain why Gene set D is not effective in identifying all of the developmentally important genes; it probably contains only a few false positives.

### ABC-transporters involved in prestalk/prespore cell differentiation

**Figure 4:**
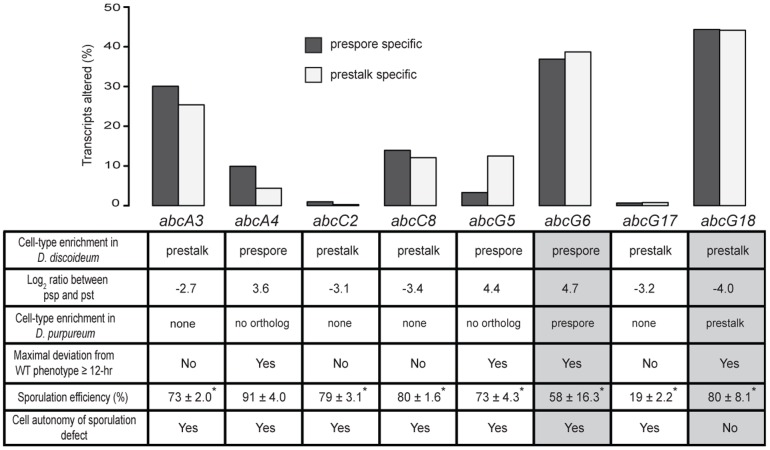
Cell type enriched *abc*-transporter transcripts and their effect on cell-type differentiation. The bar graph shows the fraction (%) of prespore- or prestalk- enriched transcripts that were altered in each of the mutants compared to the wild type. The names of the mutated *abc* genes are indicated below the bars. The table below the graph summarizes properties of the *abc*-transcripts in wild-type cells (first three rows) and the phenotypes of the respective mutant strains (last three rows). Sporulation efficiency is shown as the fraction (%) of the cells that made spores +/− S.E.M.; * indicates statistically significant difference from AX4 (Student's t-test, two sided, n = 3, p-value<0.005).

*Dictyostelium* development and cell-type differentiation are mediated, in part, by soluble signals such as cAMP, DIF and SDF [40]–[42], but little is known about the mechanisms that export these and other signaling molecules across the plasma membrane. ABC transporters are potential exporters of signaling molecules, so we were interested in identifying *abc*-transporter mRNAs that showed differential abundance between prespore and prestalk cells. We have previously identified genes that are differentially expressed between prespore and prestalk cells during slug migration [6]. Among those, we found 8 *abc*-transporter genes that exhibited strong cell-type enrichment (>6 fold) and were mutated in this study (Figure 4). We then looked for mutants whose transcriptomes differed most from the wild type at 12- and 18-hours and identified 4 such genes. We also found that among the *D. purpureum* orthologs of the *abc*-transporter genes, two exhibited similar cell-type preference, *abcG6* and *abcG18* (Figure 4). We propose that these genes are candidate exporters of developmental signals.

To test the role of the *abc*-transporter genes in signaling, we examined the sporulation efficiencies of these mutants in pure populations. We found that all of the mutants exhibited significantly lower sporulation efficiencies compared to the wild type, except for *abcA4^−^* (Figure 4). We then mixed the mutants with GFP-labeled wild-type cells and allowed them to develop in chimeras to test whether the sporulation defects were cell autonomous. We found that the sporulation defect of the *abcG6^−^* mutant is cell autonomous whereas the sporulation defect of *abcG18^−^* is non-cell autonomous, suggesting that the latter is involved in signaling. Furthermore, we performed differential expression analyses on each of these mutants compared to the wild type transcriptomes at 12- and 18-hr [24]. We focused on 1,328 previously identified cell-type enriched transcripts [6] and searched for transcripts that were differentially abundant (FDR <0.005 and Likelihood >0.9) either at 12-hr or at 18-hr between each mutant and the wild type. We observed that more than one third of the cell-type enriched transcripts were altered in the *abcG6^−^* and *abcG18^−^* mutant transcriptomes (37% and 44% respectively) (Table S7 and Table S8). These genes include cell-type specific markers such as the prespore genes *cotB*, *cotC*, *pspA* and *pspB* [43], [44] and the prestalk gene *ecmA* and *ecmB* [43]. Our results show that both *abcG18* and *abcG6* affect cell type proportioning and spore differentiation, possibly through export of signaling molecules.

Genes of the *abcB* family, namely *tagB, tagC and tagA*, play a critical role in development through signal processing and export [16], [17], [45]. *tagC*, which is primarily expressed in prestalk cells, plays a role in SDF-2 signaling [46], [47]. SDF-2 is a spore differentiation inducing peptide which is produced by processing of the Acyl Coenzyme A binding protein, AcbA [48]. SDF-2 and GABA-2 signaling result in presentation of the protease domain of TagC on the cell surface of prestalk cells, where it processes a diffusible AcbA protein, primarily produced in prespore cells, into SDF-2. The ABC transporter activity of *tagC* is necessary for the protease domain presentation [47]. Similarly, *tagB* is involved in processing the SDF-1 precursor to SDF-1 [49] whereas *tagA* and *acbA* have a coordinated role in *D. discoideum* cell differentiation [45]. In this study we used transcriptional and genetic methods to infer a role for *abcG6* and *abcG18* in spore differentiation. We propose that *abcG18*, which is expressed in prestalk cells, may facilitate the secretion of a diffusible signaling molecule that affects sporulation of prespore cells. Examples of potential cargo molecules include cytokinins, which influence sporulation during culmination [50], but we have not tested that possibility.

Others have shown that mutating both *abcG18* and *abcG2* (*abcG18^−^/abcG2^−^*) can suppress the developmental arrest and the aberration in prespore/prestalk cell ratio observed in the *rtoA^−^* mutant [51]. In this report we found that mutating *abcG18* alone leads to compromised spore differentiation. It is interesting that we did not observe other *abc*-transporter mutants that share a physiological function with either *abcG18^−^* or *abcG2^−^*, as no other mutant showed similar phenotypes to these two mutants (Figure 1 and Table 1).

## Supporting Information

Figure S1 **Each panel describes the transcript abundance of an** ***abc*** **-gene (as indicated above the panel) in the wild type (red, normalized read count) and the maximal deviation of the respective** ***abc*** **-mutant phenotype from the wild-type phenotype (blue, 1 – Spearman correlation between the wild type and the mutant) as a function of time (hours).**
(PDF)

Table S1 **This table contains information regarding the** ***abc*** **-mutant generation.** Column B contains the name of each mutant strain. Column C describes which of the two methods was used to construct the DNA vector for generating the insertional mutant: Transposon mediated mutagenesis (TZ) or direct cloning using the vector DT2-bsr (DT2). The restriction endonucleases used to clone the BSR cassette into the gene fragment in the second method are listed in column D. Columns E and F contain the primers used to amplify a genomic DNA fragment from each gene. Column G lists the insertion site of the BSR cassette within the gene relative to the ORF and the total ORF length is listed in column H.
(XLSX)

Table S2 **This table contains the oligonucleotide sequences used to determine the abundance of each** ***abc*** **-transporter transcript by quantitative RT-PCR.** The first column represents the gene name, the second column represents the forward primer and the third column represents the reverse primer.
(XLSX)

Table S3 **This table contains the GEO Accession Numbers, sample descriptions and hyperlinks of all the RNA-sequencing data deposited in the NCBI GEO public database.** Access to the entire dataset is available at: http://www.ncbi.nlm.nih.gov/geo/query/acc.cgi?token=xbuzhssekieyyxm&acc=GSE45555
(XLSX)

Table S4 **This table describes the change of all the** ***abc*** **-transporter transcript abundance in wild-type cells under various conditions determined by quantitative RT-PCR.** Treated samples were compared to untreated samples; we considered transcripts that exhibited a two fold change between the treated and untreated samples as differentially responsive. The response is represented as a binary value in the table, which is further explained in column G.
(XLSX)

Table S5 **This table contains the list of predicted developmental genes (gene set C).** The first column header ‘ddb_g’ is the primary, unique identifier for each gene in dictyBase and the second column header ‘genename’ is the primary gene name. Each baySeq comparison performed with two pre-defined models (DE-differentially expressed and NDE-not differentially expressed) at each time point (0-, 6-, 12- or 18-hr) produces two output parameters: Likelihood (Lik) and False Discovery Rate (FDR). We performed three kinds of comparisons: 1) regular mutant transcriptomes vs. wild type (reg), 2) delayed mutant transcriptomes vs. wild type (del), and 3) non developing mutant transcriptome vs. wild type (nod) as described in the text. Hence the column header ‘Lik.NDEreg00hr’ represents the Likelihood of a gene being not differentially expressed when regular mutant transcriptomes were compared to wild type transcriptome at 0-hours. The other column headers follow the same logic.
(XLSX)

Table S6 **This table contains the list of predicted conserved developmental genes (gene set D).** The column headers are similar to the ones given in Table S6.
(XLSX)

Table S7 **This table contains differential abundance data of known prespore- and prestalk- enriched transcripts in** ***abcG6^−^*** **.** The column headers are similar to the ones given in Table S6 except that in this case only *abcG6^−^* was compared with the wild type at 12- and 18-hours. The column header ‘cell_type’ indicates whether transcripts of a gene are preferentially enriched in either pre-stalk or pre-spore cell types as described in Parikh *et. al.* (2010).
(XLSX)

Table S8 **This table contains differential abundance data of known prespore and prestalk enriched transcripts in** ***abcG18^−^*** **.** The column headers are similar to the ones given in Table S8.
(XLSX)
