# Supplementary figures and images for: ABC Transporters in Dictyostelium discoideum Development
Source: PLoS One. 2013 Aug 14;8(8):e70040. doi: 10.1371/journal.pone.0070040 (PMC3743828; doi:10.1371/journal.pone.0070040)

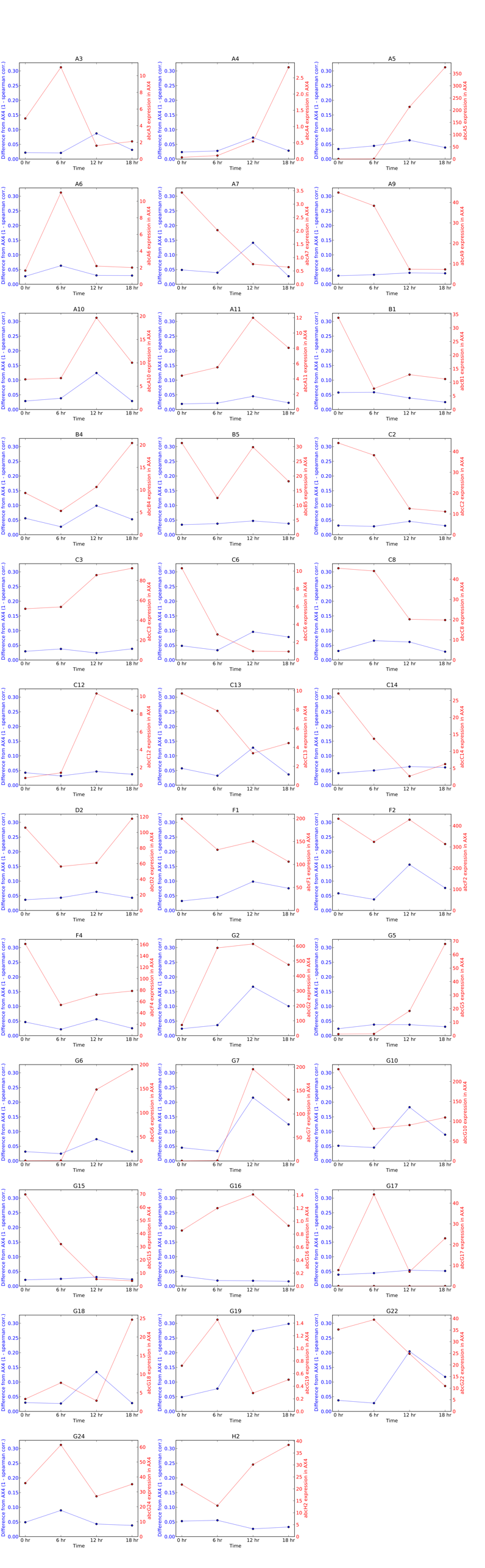

Supplement: Figure S1 — Each panel describes the transcript abundance of an abc -gene (as indicated above the panel) in the wild type (red, normalized read count) and the maximal deviation of the respective abc -mutant phenotype from the wild-type phenotype (blue, 1 – Spearman correlation between the wild type and the mutant) as a function of time (hours). (PDF) [file pone.0070040.s001.pdf]
